# Iroquois transcription factor *irx2a* is required for multiciliated and transporter cell fate decisions during zebrafish pronephros development

**DOI:** 10.1038/s41598-019-42943-y

**Published:** 2019-04-23

**Authors:** Amanda N. Marra, Christina N. Cheng, Basma Adeeb, Amanda Addiego, Hannah M. Wesselman, Brooke E. Chambers, Joseph M. Chambers, Rebecca A. Wingert

**Affiliations:** Department of Biological Sciences, Center for Stem Cells and Regenerative Medicine, Center for Zebrafish Research, University of Notre Dame, Notre Dame, IN 46556 USA

**Keywords:** Pattern formation, Organogenesis

## Abstract

The genetic regulation of nephron patterning during kidney organogenesis remains poorly understood. Nephron tubules in zebrafish are composed of segment populations that have unique absorptive and secretory roles, as well as multiciliated cells (MCCs) that govern fluid flow. Here, we report that the transcription factor *iroquois 2a* (*irx2a*) is requisite for zebrafish nephrogenesis. *irx2a* transcripts localized to the developing pronephros and maturing MCCs, and loss of function altered formation of two segment populations and reduced MCC number. Interestingly, *irx2a* deficient embryos had reduced expression of an essential MCC gene *ets variant 5a (etv5a)*, and were rescued by *etv5a* overexpression, supporting the conclusion that *etv5a* acts downstream of *irx2a* to control MCC ontogeny. Finally, we found that retinoic acid (RA) signaling affects the *irx2a* expression domain in renal progenitors, positioning *irx2a* downstream of RA. In sum, this work reveals new roles for *irx2a* during nephrogenesis, identifying *irx2a* as a crucial connection between RA signaling, segmentation, and the control of *etv5a* mediated MCC formation. Further investigation of the genetic players involved in these events will enhance our understanding of the molecular pathways that govern renal development, which can be used help create therapeutics to treat congenital and acquired kidney diseases.

## Introduction

The vertebrate kidney is an architecturally intricate organ composed of nephron functional units that cleanse the circulation of metabolic waste and maintain fluid homeostasis^1^. Nephrons contain a blood filter, a segmented epithelial tubule, and collecting duct^2^. The blood filter disperses fluid across sieve comprised of a fenestrated network of capillaries that are situated within a specialized basement membrane that is opposed by podocytes with interdigitating cellular extensions^3^. Once inside the tubule, the renal filtrate passes by a series of specialized proximal and distal epithelial segment populations that are responsible for step-wise nutrient reabsorption and metabolite secretion. Fine-tuning of salt balance and final excretion occurs through the distal segments and subsequent collecting duct.

While the composition and precise organization of nephron regions is essential for proper kidney physiology, the developmental processes that control formation of cell types are not yet fully understood^4,5^. In part, this is due to the complicated nature of kidney organogenesis, which involves the coordinated creation of nephrons that each contains a multitude of differentiated cell types in precise arrangements. For example, in its final postnatal form, a single human kidney can contain hundreds of thousands to upwards of 1.5 million nephrons comprised of over 20 differentiated cell types^6^. Further, multiple kidney forms are made during development, which involves the successive formation and degradation of several nephron-based organs. In mammals, the adult metanephros is preceded by a transitory structure termed the mesonephros, which develops after the embryonic pronephros arises from the intermediate mesoderm (IM)^7^. In fish, the mesonephros is the adult kidney and a simple pronephros functions during embryonic stages^7^. Despite these differences, the fundamental genetic and molecular composition of nephrons is conserved across vertebrate species based on the common expression of transcription factors and solute transporters that define the various differentiated cell compartments^8,9^.

In recent years, the zebrafish pronephros has emerged as a powerful model of vertebrate renal ontogeny^10,11^ and disease^12–14^ because of its rapid development and nephron conservation. During the initial stages of nephrogenesis, renal progenitors arise from bilateral stripes of IM precursors^15^ and have been characterized by dynamic spatiotemporal gene expression patterns that are modulated at the outset by retinoic acid (RA) signaling^16–18^. Following a mesenchymal-to-epithelial transition (MET) of the renal progenitors^19,20^, the zebrafish pronephros consists of two parallel nephrons that are connected caudally by a common collecting duct, and will later undergo morphogenesis events rostrally to form a shared blood filter apparatus^21,22^. The epithelial tubule of the nephron is subdivided into functional regions known as the proximal convoluted and straight tubule segments (PCT, PST) and the distal early and late (DE, DL) segments, the latter where the associated corpuscle of Stannius (CS) gland arises^16^. Each of these segments is comprised of unique differentiated transporter cells that possess a single primary cilium, which is a microtubule-based projection that extends from the apical surface into the extracellular space. Additionally, a population of multiciliated cells (MCCs) is dispersed in the intermediate region of the nephron corresponding to the PST and parts of the adjacent PCT and DE segments^23,24^. Furthermore, all cilia within the zebrafish kidney are motile and participate in the movement of filtrate through the nephron tubule^25^. Interestingly, while MCCs are a major renal epithelial cell type in zebrafish, analogous MCCs in humans have only been documented in the fetal kidney^26,27^ and in case reports of kidney diseases such as hypercalcemia, congenital nephrosis and glomerulonephritis^27–34^. These findings suggest that understanding the mechanisms that guide MCC formation may provide insights about kidney development and disease.

A growing list of genes and signaling pathways have been identified as participants in the networks that direct segmentation and MCC development during zebrafish renal organogenesis. RA both controls proximo-distal segment pattern and promotes MCC development by inhibiting expression of the *mds1/evi1 complex (mecom)* transcription factor^35^. *mecom* subsequently promotes Notch signaling which negatively modulates a binary transporter cell versus MCC fate choice^23,24,35^. In part, Notch signaling inhibits expression of the *etv5a* transcription factor, while RA promotes *etv5a* to stimulate MCC development^36^. Moreover, *etv5a* and *etv4* were found to have redundant functions since their combined deficiency caused a significantly greater decrease in MCC number than the knockdown of either factor alone^36^. These studies indicate that the MCC developmental pathway must be highly regulated, as the loss of any single factor did not completely impair MCC formation^35,36^. Consequently, the exact mechanisms that direct the specification of MCC remains are not yet completely elucidated.

The *iroquois (iro/Irx)* genes encode transcription factors that belong to the TALE superclass of homeodomain proteins and regulate the patterning of tissue territories during embryogenesis in invertebrates and vertebrates, respectively^37,38^. Several *Irx* genes are expressed during kidney development in amphibians, mammals and zebrafish^17,39–43^. For example, *irx1/2/3* transcripts are localized to the intermediate tubule region of the *Xenopus* pronephros^41^, and in the mouse metanephros are located in the intermediate region in the S-shaped body of developing nephrons and later to the middle section of the nephron, known as the Loop of Henle^41,44,45^. Knockdown of Irx2 did not alter pronephros tubule development in the frog embryo, however, leading to the hypothesis that it shares redundant activities with Irx1 and/or Irx3^41^. Interestingly, the zebrafish homologs, *irx2a* and *irx3b*, are expressed in the central region of the pronephros tubule, in a domain which includes the PST and DE segments along with MCCs^17,40^. *irx3b* is required for DE specification, where the loss of this transcription factor results in the abrogation of *slc12a1*^+^ tubule cells, expanded proximal segments, and an expanded CS lineage^17,46^. In contrast, the function(s) of *irx2a* during pronephros development have remained undefined until now.

Here, we report novel roles for *irx2a* in PST and DL segment development as well as MCC formation in the zebrafish pronephros. We found that *irx2a* regulates expression of *etv5a* in part to control MCC fate choice, and that this regulation occurs downstream of RA signaling. These findings provide the first account that *irx2a* coordinates nephron segmentation and MCC development, which has implications for understanding kidney organogenesis across vertebrates.

## Results

### *irx2a* is expressed in the central renal progenitor field and subsequent pronephros

**Figure 1:**
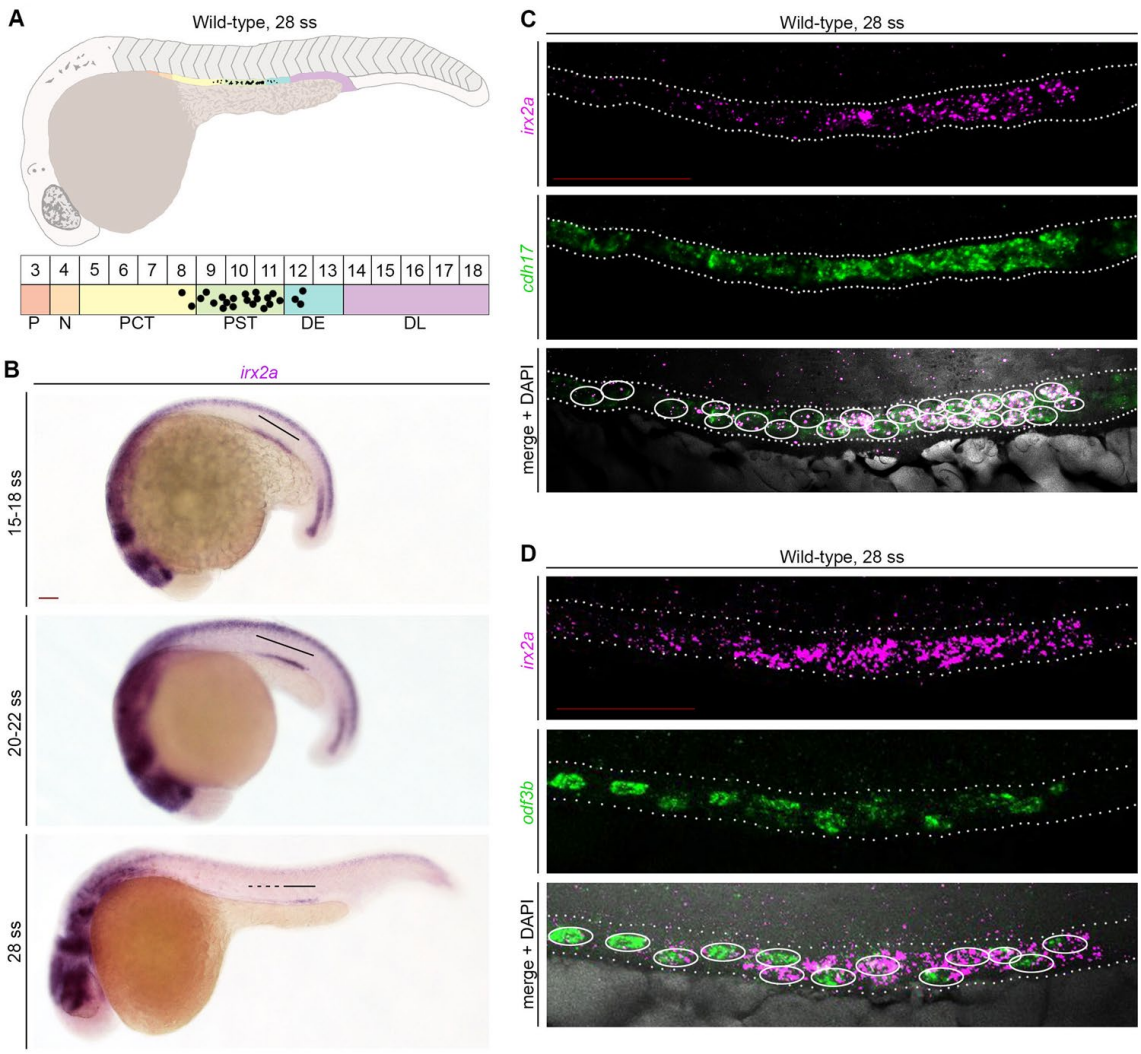
*irx2a* expression localizes to a region of the zebrafish pronephros that corresponds to the PST, DE and MCC domains. (**A**) Schematic of a zebrafish embryo (lateral view) at 24 hpf, which is equivalent to the 28 ss. Schematic below depicts color coded segments, corresponding somite numbers, and the expression pattern of MCCs in black within the nephron. MCC number is not to scale. (**B**) WISH in wild-type zebrafish embryos at the 15–18 ss, 20–22 ss, and 28 ss demonstrates *irx2a* transcript expression (purple) in the middle of the developing pronephros. Black lines highlight the *irx2a* expression domain. Scale bar is 50 μm. (**C**) FISH in wild-type embryos at the 28 ss demonstrates that *irx2a* expression (magenta) colocalizes with *cdh17*^+^ epithelial cells and (**D**) *odf3b*+ MCCs of the nephron tubule (green), where DAPI labels the nuclei (grey). Scale bar is 50 μm. White circles indicate nuclei with co-expression of the respective markers. P - podocytes, N - neck, PCT - proximal convoluted tubule, PST - proximal straight tubule, DE - distal early, DL - distal late, MCC - multiciliated cell, ss - somite stage, WISH – whole mount *in situ* hybridization, FISH – fluorescent *in situ* hybridization.

Nephron segmentation during zebrafish pronephros ontogeny is completed by the 28 somite stage (ss) and forms a series of cell types which will comprise the tubule, and others that later contribute to formation of the blood filter^16^ (Fig. 1A). The epithelial tubule populations are interspersed with MCCs, which occupy the PCT, PST, and DE segments in a “salt and pepper” like distribution^23,24^ (Fig. 1A). Further, the segment populations occupy an anatomical location in close proximity to the trunk somites, where they are situated adjacent to somites 3 through 18 (Fig. 1A). To further explore the association of *irx2a* with renal progenitor development, we performed whole mount *in situ* hybridization (WISH) on wild-type zebrafish embryos between the 5–28 ss to assess its spatiotemporal expression domain in the emerging kidney. *irx2a* transcripts were detected first at the 15 ss in a pattern consistent with the central region of the developing pronephros tubule, where *irx2a* transcripts continued to be expressed at the 20–22 and 28 ss (Fig. 1B). Next, we performed a series of double whole mount fluorescent *in situ* hybridization (FISH) experiments to further define the occupancy of *irx2a* transcripts within renal progenitors. Prior studies have shown that *cadherin17* (*cdh17*) expression demarcates the nephron tubule at the 28 ss^47^, while *outer dense fiber of sperm tails 3B* (*odf3b*) demarcates MCCs^23^. We found that *irx2a* transcripts localized both to *cdh17*^+^ and *odf3b*^+^ nephron cells (Fig. 1C,D), confirming pronephros-specific expression of *irx2a*. There was substantial overlap between cells that expressed both *irx2a* and *odf3b* transcripts (Fig. 1D). Interestingly, some *irx2a*^+^ cells were not *odf3b*^+^, and visa versa; additionally, we noted that some cells showed very low co-expression of these markers (Fig. 1D). As the onset of *odf3b* expression in the pronephros occurs at approximately the 20 ss of embryogenesis^36^, and *irx2a* was detected as early as the 15 ss, this may suggest that *irx2a* marks MCC precursors and diminishes as MCC fate is selected and/or as MCCs differentiate. Therefore, we next explored whether *irx2a* has roles in nephrogenesis during events such as segmentation and MCC ontogeny.

### *irx2a* deficiency alters PST and DL segment development in the pronephros

To explore the functional role of *irx2a* during kidney development, we conducted knockdown studies by utilizing a morpholino (MO) that specifically targeted the splicing boundary between intron 1–2 and exon 2 of the *irx2a* pre-mRNA sequence^48^ (Fig. S1A). Through RT-PCR and sequencing analysis, we confirmed that the majority of *irx2a* transcripts were misspliced in *irx2a* morphants compared to wild-type embryos (Fig. S1B,C). The aberrantly spliced *irx2a* transcript is predicted to produce a truncated peptide that lacks the homeobox domain due to inclusion of intron 1, which leads to a premature stop codon shortly after exon 1 (Fig. S1D).

**Figure 2:**
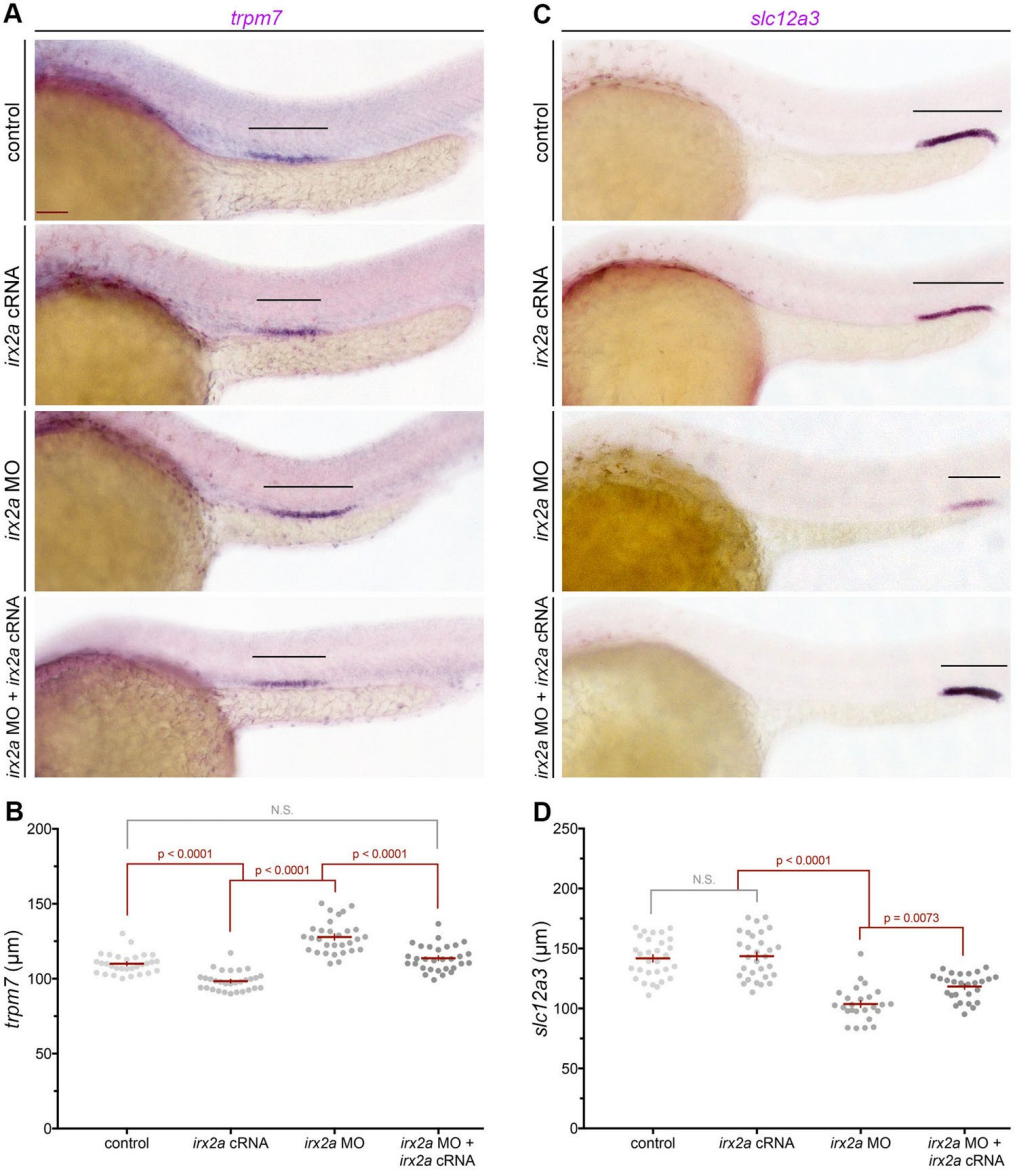
*irx2a* restricts the PST and promotes the DL during pronephros segmentation. (**A**) Lateral view of WISH analysis with the PST marker *trpm7* at the 28 ss. Black bars highlight the *trpm7* expression domain in the pronephros. Scale bar is 50 μm. (**B**) Quantification of *trpm7* length (μm). Each dot represents one nephron and data is presented +/− SEM. Statistical significance was determined with ANOVA. (**C**) WISH in 28 ss embryos with the DL marker *slc12a3* shown in a lateral view. Black bars denote *slc12a3* expression. (**D**) Quantification of *slc12a3* domain length (μm), where each dot represents one nephron. Data is presented +/− SEM and statistical significance was determined by ANOVA. WISH – whole mount *in situ* hybridization, ss – somite stage, PST – proximal straight tubule, DL – distal late tubule.

Next, we performed WISH to characterize the effect of *irx2a* deficiency on pronephros development. Using the somites, marked by *smyhc1*, as a point of reference to demarcate distinct segment boundaries, we analyzed each segment based on the specific expression of solute transporter genes: *slc20a1a* (PCT), *trpm7* (PST), *slc12a1* (DE), and *slc12a3* (DL)^16^. *irx2a* deficient embryos formed PCT and DE segments that were indistinguishable in absolute length from wild-type controls (Fig. S2). However, the position of the DE was shifted posteriorly with respect to the trunk, where it was situated adjacent to somites 13–14 compared to the wild-type location adjacent to somites 12–13 (Fig. S2). Further, *irx2a* deficient embryos exhibited an expanded PST segment and a reduced DL segment (Figs 2, S2). Measurement and statistical analysis of the showed that these segment alterations in *irx2a* deficient embryos were significant (Fig. 2B,D). To assess whether morpholino toxicity could affect pronephros development, we examined segment development by WISH following microinjection of a standard control morpholino, and observed no alteration to segment pattern formation (Fig. S3).

To explore the *irx2a* morphant phenotype further, and next test the MO specificity for these renal phenotypes, rescue studies were conducted in *irx2a* deficient embryos by provision of *irx2a* capped mRNA (cRNA). Expression of *irx2a* was sufficient to rescue both PST and DL development in *irx2a* deficient embryos, which was found to be statistically significant based on measurement of absolute segment lengths (Fig. 2). Additionally, *irx2a* overexpression studies were performed in wild-type embryos to assess whether *irx2a* cRNA was sufficient to induce alterations in the PST or DL lineages. However, at *irx2a* cRNA dosages that did not grossly affect embryogenesis, there was no significant difference observed in the populace of either the PST or DL segments (Fig. 2). Taken together, these results indicate that *irx2a* is necessary but not sufficient for PST and DL segment fate during pronephros development.

### *irx2a* deficiency alters MCC development in the pronephros

**Figure 3:**
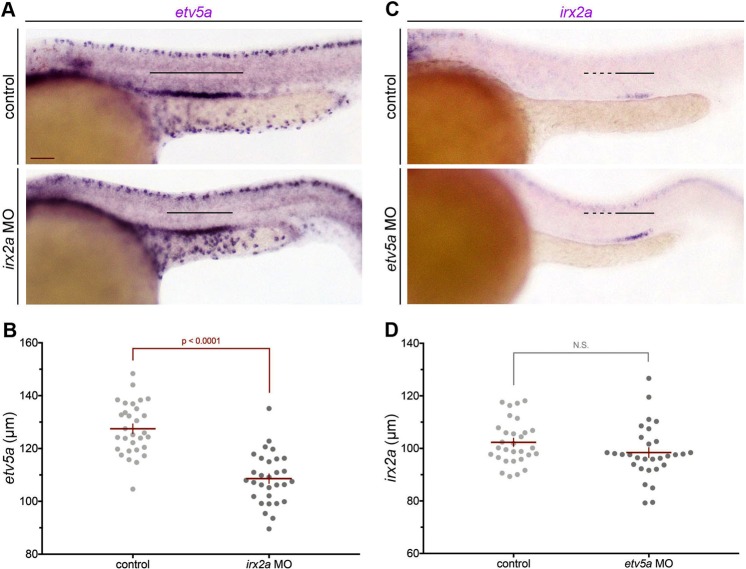
*irx2a* acts upstream of *etv5a*. WISH at the 28 ss revealed decreased *etv5a* expression in the pronephros of *irx2a* morphants. Black bars denote the *etv5a* expression domain. Scale bar is 50 μm. (**B**) Quantification of *etv5a* length in μm at the 28 ss. Each dot represents one nephron and data is presented +/− SEM. Significance was evaluated with an unpaired student’s T-test. (**C**) The *irx2a* domain is unchanged in *etv5a* morphants at the 28 ss. Solid black bars denote strong *irx2a* expression and the dashed black lines denote faint expression in the pronephros. (**D**) Quantification of *irx2a* length (μm) at the 28 ss. Each dot represents one nephron and data is presented +/− SEM. An unpaired student’s T-test was used to determine significance. WISH – whole mount *in situ* hybridization, ss – somite stage.

Given the expression domain of *irx2a* in maturing MCCs, we next explored whether MCC ontogeny in the pronephros is influenced by *irx2a* activity. MCC development is known to be reliant on expression of the transcription factor *etv5a*, which establishes the domain of MCC progenitors through interplay with Notch signaling^36^. Upon using WISH to examine *etv5a* expression in *irx2a* deficient embryos, we found that the pronephros domain of *etv5a* was significantly reduced in absolute length compared to wild-type controls (Fig. 3A,B). Next, *irx2a* expression was assessed in *etv5a* deficient embryos using WISH. We found that the pronephros expression of *irx2a* was comparable between wild-type and *etv5a* deficient embryos, where measurement of the *irx2a* expression domain within the nephron tubules confirmed that there was no statistically significant difference in length compared to wild-type embryo controls (Fig. 3C,D). Taken together, these data suggested that *irx2a* acts upstream of *etv5a* during nephrogenesis, and led us to hypothesize that *irx2a* was likely requisite for MCC fate choice.

**Figure 4:**
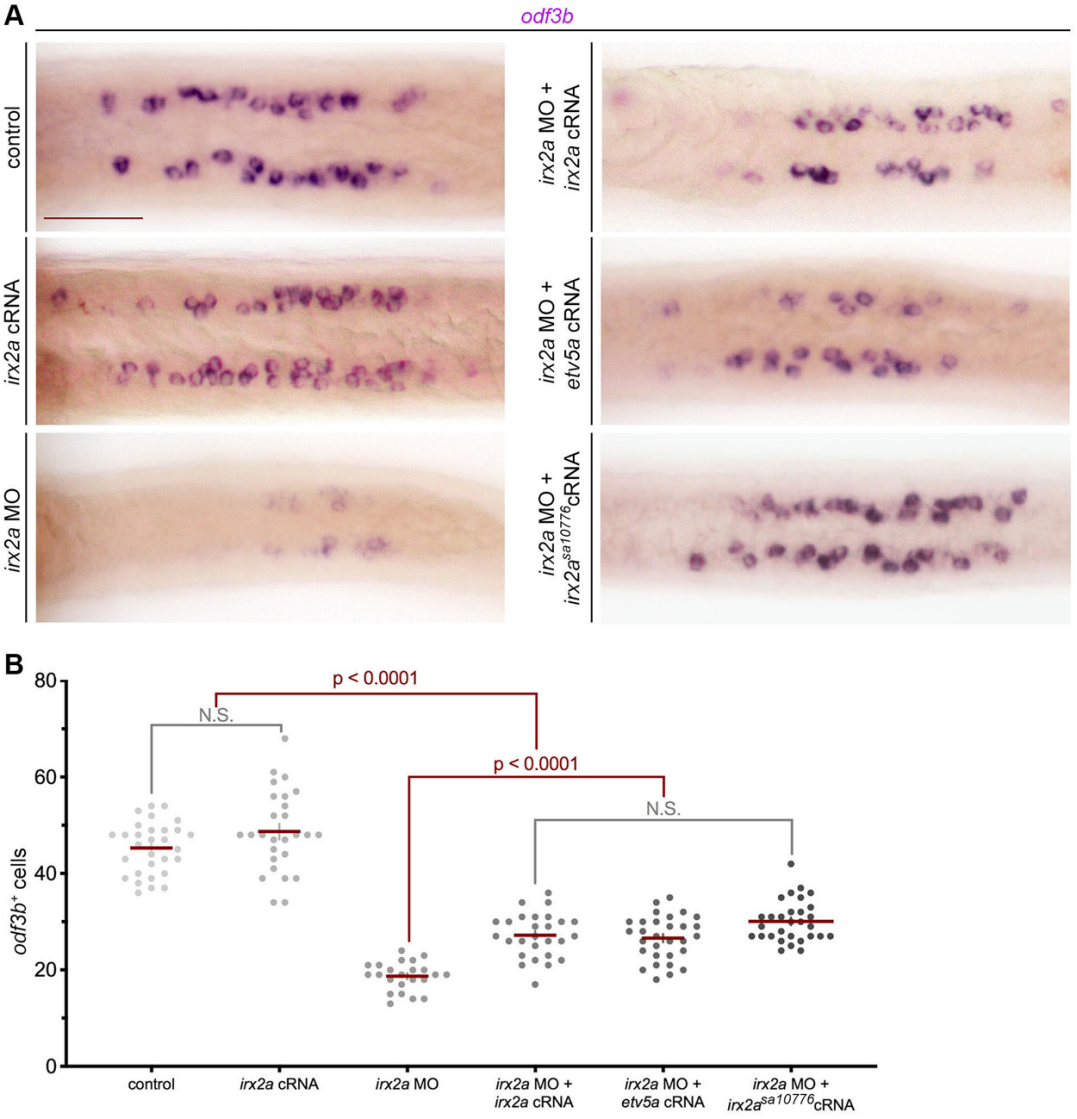
*irx2a* is required upstream of *etv5a* for MCC genesis. (**A**) Dorsal view of *odf3b* in the pronephros by WISH at the 28 ss shows a decrease in maturing MCCs in *irx2a* deficient embryos, which was rescued with *irx2a* cRNA, *etv5a* cRNA, and *irx2a*^*sa10776*^ cRNA. Scale bar is 50 μm. (**B**) Quantification of *odf3b*^+^ MCCs at the 28 ss. Each dot represents one pronephros. Data is presented +/− SEM and statistical significance was determined with ANOVA. WISH – whole mount *in situ* hybridization, ss – somite stage, MCC – multiciliated cell.

To explore this notion, MCC development in the pronephros was assessed in *irx2a* gain and loss of function studies (Fig. 4). WISH was performed to evaluate *odf3b*^+^ cell number as this marker specifically labels maturing MCCs^23^. Overexpression of *irx2a* was not associated with a significant increase in absolute MCC number compared to wild-type controls (Fig. 4A,B). However, *irx2a* deficient embryos developed significantly fewer numbers of nephron MCCs compared to wild-type controls or *irx2a* overexpressing cohorts at the 28 ss (Fig. 4A,B). Similarly, reduced MCC numbers were observed at 36 hpf in *irx2a* deficient embryos, likely latter ruling out a phenotype attributable to developmental delay (Fig. S4). Next, we found that the reduction of nephron MCC number in *irx2a* deficient embryos was partially rescued by provision of *irx2a* cRNA (Fig. 4A,B).

In light of the observation that *irx2a* deficiency led to reduced *etv5a* expression, and prior knowledge that the latter is essential for MCC fate choice^36^, we subsequently tested if *etv5a* could rescue MCC ontogeny in *irx2a* morphants. Provision of *etv5a* cRNA in *irx2a* deficient embryos partially rescued MCC number, which was statistically significant compared to *irx2a* knockdown alone, and statistically equivalent to MCC rescue following *irx2a* cRNA expression (Fig. 4A,B). Based on these results, we conclude that *irx2a* is essential for MCC fate where it acts upstream of *etv5a* in a shared pathway that promotes MCC development in renal progenitors.

### Phenotypic analysis of an *irx2a* genetic mutant

**Figure 5:**
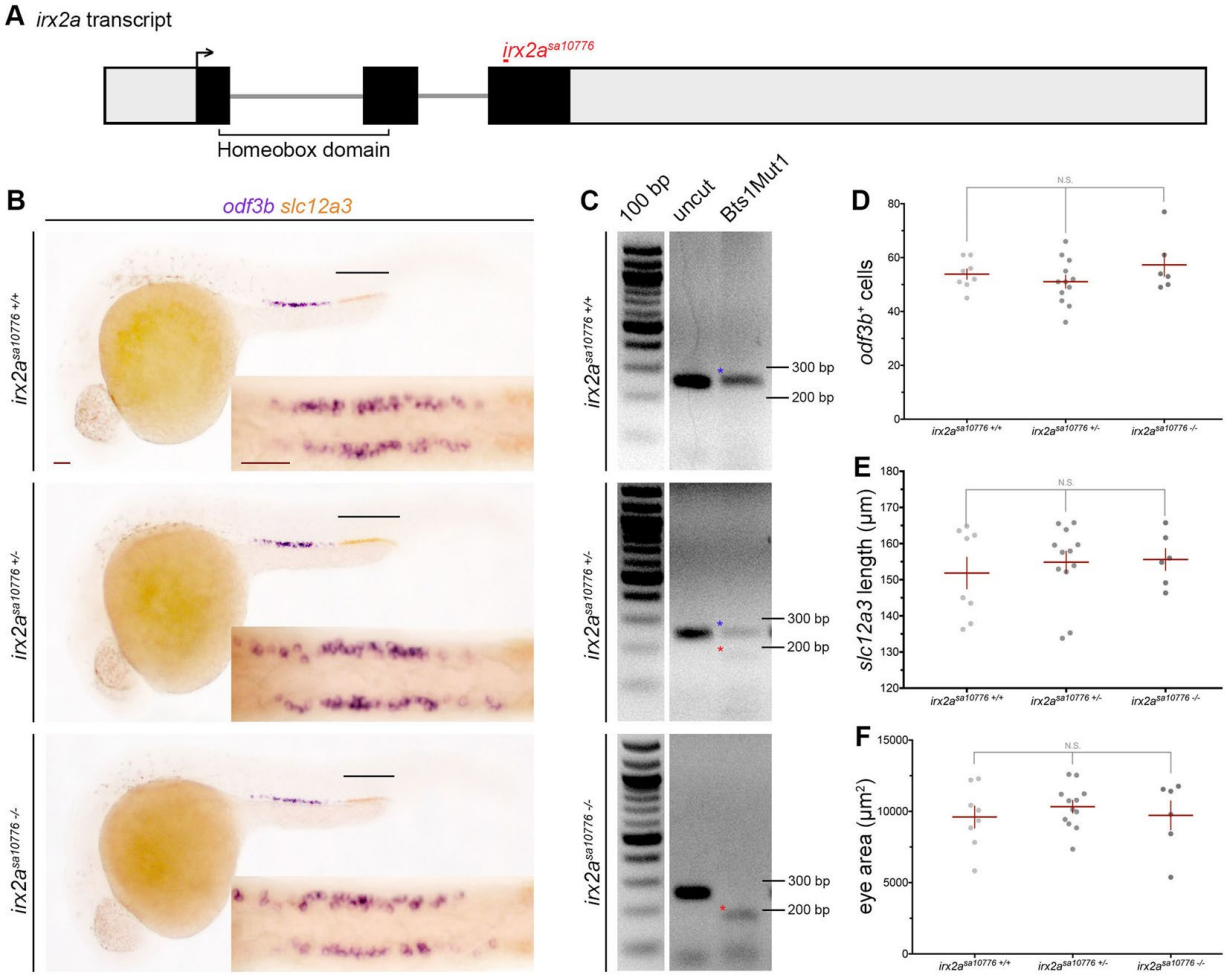
*irx2a* genetic mutants do not have a renal or eye phenotype. (**A**) Schematic of the *irx2a* transcript and location of the *irx2a*^*sa10716*^ mutation, where grey is non-coding and black represents coding sequence. The forward arrow demarcates the ATG start site. (**B**) WISH analysis at the 28 ss shows *odf3b* and *slc12a3* expression in *irx2a*^*sa10716*+/+^, *irx2a*^*sa10716*+/−^, and *irx2a*^*sa10716*−/−^ embryos. The black lines highlight the DL marker *slc12a3*. An enlarged dorsal view of the *odf3b*+ MCCs for each embryo is shown in the inset. Scale bars are 50 μm. (**C**) Gel image of the restriction digest product for each genotype. Wild-type bands are marked with a blue star, and mutant bands with a red star. (**D**) Quantification of *odf3b*+ cells at the 28 ss. Each dot represents one pronephros. (**E**) Quantification of the *slc12a3* length (μm) at the 28 ss. Each dot represents one nephron. (**F**) Quantification of eye area (μm^2^). Each dot represents one eye. For all graphs, data is presented +/− SEM and statistical significance was determined by ANOVA. WISH – whole mount *in situ* hybridization, ss – somite stage, DL – distal late, MCC – multiciliated cell.

To perform further studies of the roles of *irx2a* in development, we obtained the *irx2a*^*sa10776*^ line from the Zebrafish International Resource Center, which was generated by the Zebrafish Mutation Project^49^. The *irx2a*^*sa10776*^ line was reported to harbor a C- > T mutation that encodes a nonsense mutation, which is predicted to produce a truncated protein that contains only 260 of the 432 amino acids that are present in the wild-type protein (Fig. 5A). We developed a restriction fragment length polymorphism genotyping assay that utilized digestion of a PCR product to confirm and identify the mutant allele. Adult *irx2a*^*sa10776*^ heterozygotes were identified and incrossed to collect clutches for analysis of renal progenitor development using WISH followed by genotyping. Interestingly, *irx2a*^*sa10776*^ homozygote and heterozygote embryos displayed both normal nephron segmentation and MCC development (Fig. 5B–D). Assessment of MCC number through quantification of *odf3b*^+^ cells showed that there were no significant differences between wild-type, *irx2a*^*sa10776*^ heterozygote and homozygote embryos (Fig. 5B–D). Assessment of DL segment development based on the absolute domain length of *slc12a3*^+^ cells also showed no significant differences between these groups (Fig. 5B–D). Eye development was normal as well based on gross morphology and size, in contrast to a role for *irx2a* in this organ^48^ (Fig. 5E). Other studies have found inconsistencies between knockdown and mutant phenotypes due to genetic compensation in the latter^50^. Thus, *irx2a*^*sa10776*^ mutants may have normal renal progenitor development due to mechanisms that otherwise compensate for the loss of *irx2a* within the genome. Alternatively, it is also possible that the location of this nonsense mutation is not sufficient to interrupt Irx2a function.

To explore the latter, we performed site-directed mutagenesis on our *irx2a* expression construct to modify the sequence so that it would encode the *irx2a*^*sa10776*^ allele. Following synthesis of *irx2a*^*sa10776*^ cRNA and microinjection into *irx2a* morphants, MCC development was assessed by WISH for expression of *odf3b* transcripts. Interestingly, we found that *irx2a*^*sa10776*^ was sufficient to rescue MCC number comparable to *irx2a* cRNA (Fig. 4A,B). Given this result, and as the nonsense mutation in *irx2a*^*sa10776*^ is situated after the homeobox domain, we concluded that the mutant protein in fact retains the key functional attribute(s) necessary to support pronephros development. Thus, the normal appearance of renal MCCs and segments in *irx2a*^*sa10776*^ mutants is not likely due to genetic compensation by other factors.

### RA signaling is an upstream regulator of *irx2a* during nephrogenesis

We next sought to determine the relationship between *irx2a* and RA signaling to explore how *irx2a* further fits within the genetic cascades that regulate pronephros segment and MCC development. RA is a diffusible morphogen that is necessary to establish rostrocaudal patterning among pronephros progenitors during the earliest stages of nephrogenesis, and subsequently modulates the expression of many transcription factors to direct segment formation^16,17^. Furthermore, treatment with exogenous all-trans RA results in an expansion of proximal segment identities including MCCs at the expense of the distal segment regions^16,35^. Conversely, the inhibition of RA signaling by blocking its biosynthesis with the inhibitor N,N-diethylaminobenzaldehyde (DEAB) causes distal fates to be favored, while decreasing proximal lineages and abrogating MCCs^16,35^.

**Figure 6:**
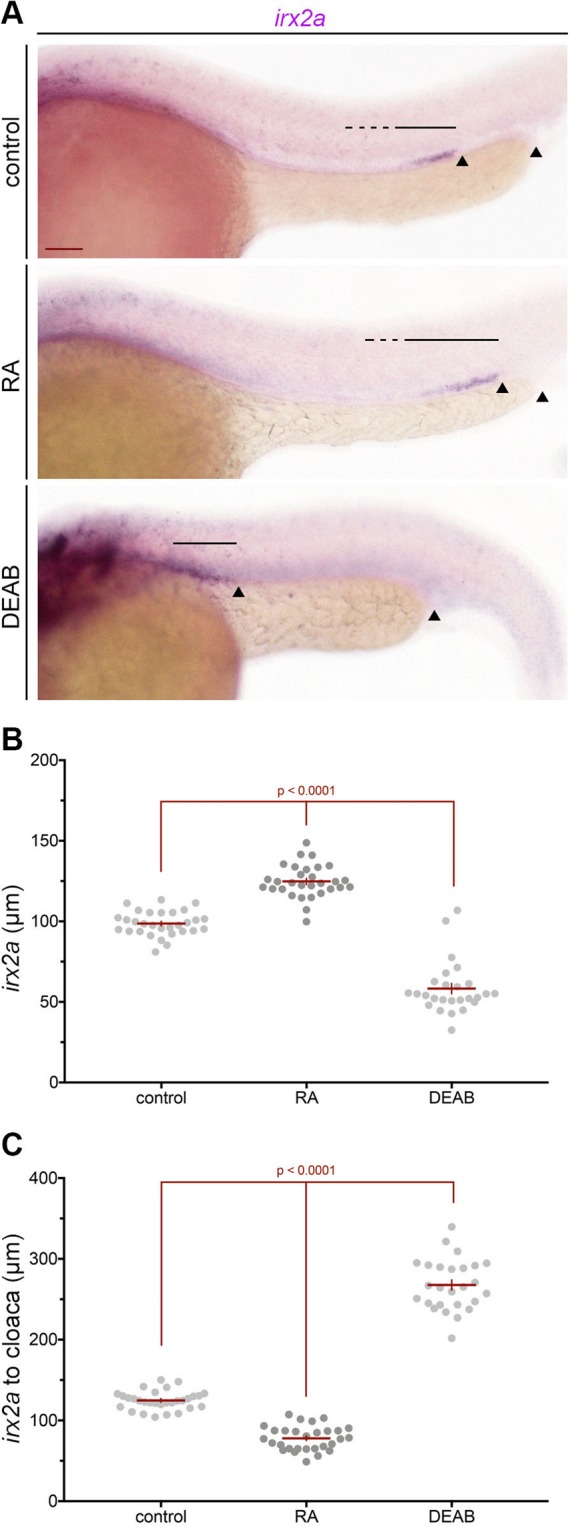
RA signaling regulates *irx2a* expression during nephrogenesis. (**A**) WISH analysis at the 28 ss demonstrates alterations in the *irx2a* pronephros domain after either treatment of RA or DEAB. The black arrowheads show the distance from distal *irx2a* expression to the cloaca, and the black lines highlight *irx2a* expression. (**B**) Quantification of *irx2a* length (μm) at the 28 ss. Each dot represents one pronephros. (**C**) Quantification of the length from *irx2a* expression to the cloaca (μm), where each dot represents one pronephros. For each graph, data is represented +/− SEM and significance was determined with an ANOVA. WISH – whole mount *in situ* hybridization, ss- somite stage, RA – retinoic acid, DEAB - N,N-diethylaminobenzaldehyde.

Therefore, we tested whether alterations in RA levels affect *irx2a* expression within the pronephros. Wild-type embryos were treated with RA or DEAB at 60% epiboly and fixed at the 28 ss for WISH analysis. We found that treatment of wild-type embryos with exogenous all-trans RA resulted in a domain expansion and a distal shift of *irx2a* expression (Fig. 6). Alternately, treatment with DEAB caused a reduction and concomitant proximal shift of the *irx2a* expression domain in the pronephros (Fig. 6). These results indicate that RA controls the expression of *irx2a* either directly or indirectly during renal ontogeny, which suggests *irx2a* acts downstream of RA to pattern the PST, DE, and MCCs.

## Discussion

**Figure 7:**
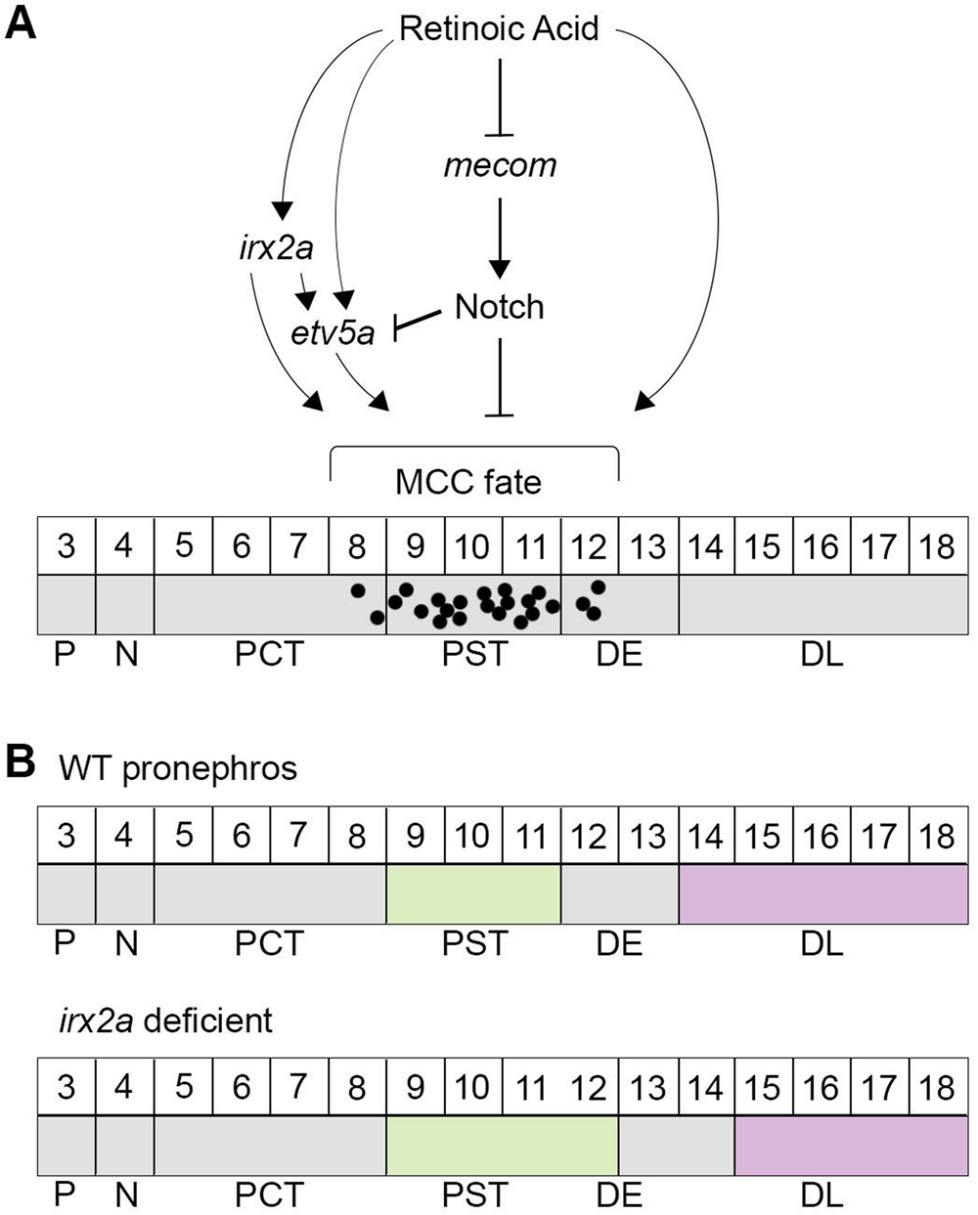
Model of *irx2a* function during segmentation and MCC specification during zebrafish nephrogenesis. (**A**) Renal MCC ontogeny is known to be reliant on RA signaling which acts upstream of the transcription factors *mecom* and *etv5a*, where negative regulation by Notch signaling restricts MCC fate choice. The present work adds to this model by demonstrating for the first time that *irx2a* functions downstream of RA signaling to regulate the expression of the *etv5a* transcription factor, thereby directing MCC fate. *irx2a* may act directly or indirectly on *etv5a*. *irx2a* likely acts on other targets as well to control MCC fate. (**B**) Diagram summarizing wild-type and *irx2a* morphant nephron segments with respect to their somite boundaries. MCC - multiciliated cell, P - podocytes, N - neck, PCT - proximal convoluted tububle, PST - proximal straight tubule, DE - distal early, DL - distal late, WT – wild-type.

There is an increasing appreciation of the genetic factors that control nephrogenesis processes across vertebrate species^9,51–57^. However, the molecular mechanisms that direct the specification of renal progenitors into the distinct cell populations that comprise each nephron segment is still not completely understood. Further defining the genetic mechanisms that govern renal organogenesis has direct relevance to deepening our knowledge about the mechanisms of kidney disease and regeneration^58,59^. The present work has enhanced our understanding of the renal transcription factor code that currently defines the zebrafish pronephros by defining several essential roles of *irx2a* within the regulatory networks that guide renal progenitor fate decisions (Fig. 7). Previous research has established that RA acts upstream of *mecom* and Notch signaling to influence MCC renal development in the zebrafish embryo, where *etv5a* is necessary for MCC fate downstream of RA^35,36^. In the present report, our results indicate that RA signaling regulates *irx2a* expression, either directly or indirectly, in the developing nephron, where *irx2a* is necessary for a normal balance of PST, DL and MCC development (Fig. 7). Given that *irx2a* transcripts are not expressed in the DL, it is intriguing that the knockdown of *irx2a* expanded the PST at the expense of the DL. Thus, it is possible that the effect on the DL may be an indirect consequence of altered proximal segment development in *irx2a* deficient embryos. With regard to MCC genesis, our results here are consistent with the conclusion that *irx2a* regulates *etv5a* during the specification of MCCs (Fig. 7). Again, the present study does not resolve whether these interactions are direct or indirect (Fig. 7). However, it is likely that *irx2a* influences additional targets during MCC genesis, as *etv5a* was only partially able to rescue MCC numbers in *irx2a* deficient embryos.

Three ancestral *Irx* genes—*araucan*, *caupolican*, and *mirror*—were originally identified in *Drosophila melanogaster* where they comprise the ‘Iro-C complex’ that controls segmentation of the body and regulates later formation of sense organs, the thorax, and wing vasculature^60,61^. *Irx* gene functions have been ascribed to many aspects of vertebrate organogenesis as well^37,38^, including the kidney^17,41–45,62^. During *Xenopus* embryonic kidney development, *Irx1* and *Irx3* have an early role in maintaining the pronephric territory^42^, and subsequently *Irx3* is essential for intermediate nephron tubule fate, in part by controlling expression of *Irx1* and *Irx2* in renal progenitors^41^. Interestingly, knockdown of *Irx1* or *Irx2* did not perturb nephron development, suggesting redundant functions for these genes during nephrogenesis^41^. In zebrafish, *irx3b* knockdown led to the abrogation of the DE with compensatory expansions of the PCT and PST, resulting in an overall increase in the proximal pronephros^17^. This data highlights a clear requirement for *irx3b* in DE segment development, and suggests that *irx3b* may also negatively modulate the PCT and PST segment domains, perhaps by regulating the position of the PCT-PST boundary^17^. As the current work indicates that *irx2a* negatively regulates PST size, assessment of *irx2a/3b* doubly deficient zebrafish embryos may provide further insights into segmentation mechanisms.

Further, the relationship between Irx2a and the transcription factor genes *sim1a* and *ppargc1a* will be necessary to define, as the proper balance of Sim1a/Ppargc1a is necessary to form the PST segment in the zebrafish pronephros^63,64^. With regard to the role of *irx2a* in regulating DL development: as noted above, this effect could be a secondary consequence of the PST phenotype, or could involve direct effects on distal renal progenitors. As the *emx1* transcription factor was recently found to promote the DL and to be a negative regulator of *irx3b* and *irx1a* in the DE, it will be of interest to test if and how *irx2a* interfaces with this network^65^. *irx2a* deficiency also phenocopies the segment changes (PST & DL) observed in prostaglandin signaling gain of function studies^66^, and *mecom* loss of function studies^35^. Moreover, the *tbx2a/2b* transcription factors are requisite for DL development^67^. In the zebrafish kidney, expression of four other *irx* genes has been documented as well (*irx1a* in the DE; *irx2a, irx4a*, and *irx5b* in both the PST and DE), denoting these genes as potential regulators of these segments^40,68^. Determining the relationship of *irx2a* with these various *irx* family members, and exploring the hypothesis that functional redundancy may exist between these related genes in renal progenitors, will further our understanding of the networks that underlie pronephros segmentation.

In addition to the contrast between our findings and the *Xenopus* pronephros where *Irx2* knockdown does not cause renal anomalies^41,42^, homozygous *Irx2* mutant mice are healthy and lack obvious organ deficiencies^69^. Here, *lacZ* expression faithfully recapitulated the endogenous *Irx2* domain in various tissues including the central nervous system, kidneys, pancreas, and lungs in *Irx2*^*LacZ/*+^ mice where the null allele was generated by targeted introduction of an IRES-*lacZ* reporter and neomycin resistance gene^69^. The intercross of these *Irx2*^*LacZ/+*^ mice produced progeny according to normal Mendelian ratios, and analysis of homozygous *Irx2* mutants showed that the *Irx2* transcript level was markedly reduced but did not result in protein production, signifying loss of function^69^. One explanation for the normal renal formation and function in *Irx2* knockout mice may be functional redundancy with other *Irx* family members. Indeed, *Irx1/2* were placed downstream of HNF1B during intermediate tubule development in the murine metanephros^44,45^. It is possible that evolutionary differences exist between *Irx* gene functions in zebrafish and other vertebrates including mammals^70^. Most vertebrates have six *Irx* genes that are organized into two clusters, *Irx*A and *Irx*B, arising from a duplication event that occurred early in their evolution^71^. Following teleost divergence from the tetrapod lineage, zebrafish underwent another whole genome duplication, which gave rise to four *irx* clusters and a single isolated gene locus. Of these, *irx2b* was deleted from the zebrafish genome^72–77^. Consequently, it is possible that neofunctionalization occurred where *irx2a* accrued adaptive mutations allowing it to perform unique functions during cell specification rather than dependently operating under the control of *irx3b* as the findings in the *Xenopus* studies predict^41^.

The current work implicates for the first time that an *Irx* transcription factor has essential functions in MCC development. MCCs have motile cilia and are responsible for stimulating fluid propulsion across tissues in a synchronized and directional manner^78,79^. In these MCCs, each cilium is characterized by a bundle of microtubules organized in a 9 + 2 configuration that is surrounded by a plasma membrane and anchored to the surface of the cell by a basal body^78,79^. The motility of a cilium is then stimulated by dynein arms located in between the nine microtubule doublets, resulting in a bending movement due to the action of these motor proteins^78,79^. In contrast, most cells in vertebrates contain a single, non-motile primary cilium that predominantly functions in signal transduction^78,79^. Despite the known significance of MCC function during development and homeostasis, the anatomical location of these intriguing cells in mammals throughout the brain ventricles and airways, for instance, has historically precluded them from comprehensive genetic examinations. In recent years, however, the application of functional genomics has led to numerous advancements due to the use of various animal and cell culture models^79,80^. Efforts to better define the transcriptional programs and ancillary modulating factors of MCC differentiation have established Notch signaling as an essential regulator of multiciliated fate choice^23,24,81,82^. This mechanism was found to be conserved between MCCs located in the zebrafish embryonic kidney, murine respiratory tract, and *Xenopus* epidermis, where the latter has also served as an excellent model for the live imaging of MCCs^82–85^. Furthermore, the microRNA, miR-499, was recently discovered to control the expression levels of Notch and Delta-like1, thereby contributing another layer of control to the pathways that influence MCC differentiation^86^. Additional studies in *Xenopus* and mice lead to the identification of a Notch target, Multicilin, which is a small coiled-coil protein that acts in a complex with the E2f4/5 transcription factors to regulate multiciliogenesis through the activation of key centriole replication genes^87,88^. Other research in these model organisms have identified that members of the Rfx family^89^, including Rfx2/3, C-Myb, and FoxJ1, operate in evolutionarily conserved functions downstream of Multicilin to promote the genesis of motile cilia^80,81,90,91^.

As we have now determined newfound roles for *irx2a* in MCC development (Fig. 7), *irx2a* is a promising candidate for further exploration in terms of conserved ciliary transcriptional programs. Epistasis experiments between *irx2a* and Notch signaling are necessary to understand their relationship during MCC emergence. Additionally, the expression of *rfx2, foxj1a/b, gmnc*, and *mcidasl* has been documented in the zebrafish pronephros and/or MCCs^23,24,92^. Studies should be conducted to examine possible relationships between these regulators of ciliogenesis and *irx2a*. Further, it will be valuable to ascertain whether Irx2a affects *etv5a* expression directly or indirectly, as *etv5a* was recently linked to the control of MCC differentiation by its regulation of prostaglandin signaling^93^. These genetic analyses will hold two-fold significance where they can help refine our understanding of the MCC molecular mechanisms of programming from precursor cells in the zebrafish kidney, and provide data for additional comparison of ciliary regulatory networks between species and tissues.

The importance of MCCs can be appreciated by their diverse roles in multiple human tissues without which dysfunction and disease would ensue^78,79^. With respect to the respiratory tract, MCCs promote the clearance of mucus, an important protective mechanism against pathogenic agents. Moreover, disruption of fluid depth between the mucosal layer and airway epithelium can lead to faulty mucus clearance by MCCs, and complicate respiratory diseases such as cystic fibrosis^78,79^. In the ventricles of the brain, MCCs enable the circulation of cerebrospinal fluid, while in the female reproductive system they facilitate the movement of the egg through the Fallopian tubes^78,79^. Failure of MCCs to perform these tasks increases the risk of hydrocephalus and infertility, respectively^78,79^.

In contrast, only a few isolated reports have documented the presence of MCCs in the human fetal kidney^26,27^ and in kidney disease states^27–34^—a stark contrast to the zebrafish kidney, where MCCs and the motile primary cilia on transportor cells are required for fluid propulsion^33^. It has been hypothesized that renal MCCs were evolutionarily lost due to the elevation of blood pressure in higher vertebrates, such as mammals, which negated the need for MCCs and motile primary cilia to regulate fluid flow^33,34,94,95^. Although MCCs are absent in human post-natal, healthy kidneys, their presence in the fetal kidney may suggest roles during early development. The manifestation of MCCs in human kidney disease, like nephrotic syndrome and renal sarcoidosis associated with hypercalcemia, also incites curiosity to the function of this cell type in these conditions. One hypothesis is that the kidney is reverting to a more primitive state in response to damage^34^. For example, the formation of cysts in the kidney could promote the reappearance of MCCs as a means to combat fluid accumulation. Consistent with this notion, the Foxj1 transcription network is upregluated after kidney injury in the zebrafish pronephros and in both mammalian cystic kidneys and following acute reperfusion injury^96^. The appearance of MCCs may thus constitute a regenerative response. Future study of MCC dynamics during renal regeneration in zebrafish could be used to assess their biological functions in this context as well^97^. While MCCs are differentially expressed between zebrafish and mammalian kidneys, it is likely that some degree of conservation exists between the transcriptional cascades that guide MCC formation among vertebrates. Therefore, the zebrafish pronephros is a useful setting for high-throughput analyses such as chemical screening^98^ to discover MCC relevant factors. Such future studies can advance our knowledge about ciliogenesis mechanisms, and provide insight into the etiology of ciliopathies while aiding the identification of potential therapeutic agents.

## Materials and Methods

### Zebrafish husbandry and ethics statement

Zebrafish were housed and cared for in the Center for Zebrafish Research at the University of Notre Dame Freimann Life Science Center, where the Institutional Animal Care and Use Committee approved the experiments documented here under protocol 16-025. All methods were carried out in accordance with relevant guidelines and regulations. Tübingen strain wild-type zebrafish were used, and staged as described^99,100^.

### Expression analysis and image acquisition

Whole mount *in situ* hybridization (WISH) was conducted as described^101,102^. Anti-sense RNA probes were digoxigenin-labeled (*etv5a, odf3b*, *cetn4*, *scl*, *irx2a*, *cdh17*, *slc20a1a*, *trmp7*, *slc12a1*, *slc12a3*, *jag2b*) or fluorescein-labeled (*smyhc1*, *cdh17, pax2a*), and generated by *in vitro* transcription using plasmid or PCR templates as described^16,17,36,63,103^. Embryos were mounted in glycerol and images were taken using a Nikon Eclipse Ni with a DS-Fi2 camera. Whole mount fluorescent *in situ* hybridization (FISH) was performed as described^103^ with the digoxigenin and fluorescein-labeled RNA probes used in WISH. Stains were developed with the TSA plus Cy3 and TSA plus Fluorescein kits (Perkin Elmer). Embryos were mounted in Poly Aqua-mount as described^104^ and imaged on a Nikon C2 confocal microscope. Z-stacks were processed with FIJI into max image projections, and all figures were assembled using Adobe Photoshop CS5.

### Genetic tools

An antisense morpholino (MO) was used to block *irx2a* splicingwith the following sequence^48^: 5′-ACGGAGAGCCCTTCAAAAATAAC-3′ (ZFIN Annotation MO2-irx2a) at 133 µM. The antisense standard control MO was used at was: 5′-CCTCTTACCTCAGTTACAATTTATA-3′ at 133 µM. MOs were synthesized and purified by Gene Tools, LLC (Philomath, OR), and resolubilized with DNase/RNase free water for storage at −20 °C. *irx2a*^*sa10716*^ were obtained from the Zebrafish International Resource Center^49^. For genotyping *irx2a*^*sa10716*^, we developed a restriction fragment length polymorphism assay. Genomic DNA was isolated from individual adult fins or embryos as described^105^. PCR amplification was performed with primers flanking the mutation site: 5′-GACCTTGTTTGCGATTCTGGAGCTGAAATC-3′ and 5′- GGGGTCCGAAGTGGCGATCTCTGCTAATGA-3′. The cycling conditions were as follows: initial 4 minute denaturation step at 94 °C followed by 45 cycles of 94 °C for 30 seconds, annealing for 30 seconds at 68 °C, 72 °C for 1 minute and a final extension step at 72 °C for 10 minutes. PCR products were purified using a Qiagen PCR Purification Kit and sent to the University of Notre Dame Genomics Core for sequencing. Restriction digest of the purified PCR product was performed using the enzyme Bts1Mut1 (NEB). The digest reaction (10 μL PCR product, 7 μL molecular dH2O, 2 μL CutSmart Buffer, 1 μL Bts1Mut1 enzyme) was incubated at 55 °C overnight. 10 μL of the digest product was then run on a 2% agarose gel.

### RT-PCR verification of splice-blocking morpholinos

20–30 uninjected and injected embryos at the 28 ss were homogenized in 500 μL TRIZOL (Ambion) and RNA was isolated according to manufacturer insructions. PCR amplification was performed using the SuperScript® IV First-Strand Synthesis System (Invitrogen) according to manufacturer instructions with primers to amplify the region between *irx2a* exons 2 and 3: forward 5′-GACGAAGACGAAGATGACGGAGATG-3′, reverse 5′-CTCGCATTTGTCCTGGATTTCAGCT-3′. Products were isolated by agarose gel extraction (Qiagen QIAquick Gel Extraction) and sequenced.

### cRNA synthesis and rescue experiments

The zebrafish *irx2a* open reading frame (ORF) was PCR amplified using high fidelity TAQ polymerase from the Expand PCR kit (Roche) in combination with a PCR Mix solution (100 mM dNTPs, 1 M MgCl2, 1 M Tris-HCl (pH 8.4), 4 M KCl, 1% Gelatin, 100 mg/mL BSA, and sterile H2O) and primers specific for *irx2a:* forward, 5′-**ATTC***GAATTC*GCCGCCACC atgtcctatcctcagggttacctctaccagcccccgggctc-3′ and reverse, 5′**-CGAC***CTCGAG* ttaactcgacaggtaagattgggatcttactgtgaaaacctcgctggggt-3′. The forward primers were designed with a 4 bp anchor (bold print) followed by the 6 bp EcoR1 sequence (italicized), the Kozak consensus (underlined), and finally a sequence beginning at the start site (lowercase). Alternatively, the reverse primer contains the stop sequence (lowercase), 6 bp Xho1 sequence (italicized), and a 4 bp anchor (bold print). Amplified ORF was ligated into the pCS2 vector and transformed into DH5α competent cells (Invitrogen). Mutagenesis was performed on the *irx2a*.pCS2 construct to create *irx2a*^*sa10776*^.pCS2 using the QuikChange Site Directed Mutagenesis Kit (Agilent Technologies, 200518-12) as previously described^106^. The mutagenesis primers were forward, 5′-GGTGACGCGCCTCACTGCCTTTCCTCC-3′ and reverse, 5′-GGAGGAAAGGCAGTGAGGCGCGTCACC-3′. *etv5a* was synthesized from a previously reported rescue construct template^33^. All capped RNA (cRNA) was synthesized *in vitro* using the mMESSAGE mMACHINE SP6 Transcription kit (Ambion) and stored at −80 °C. Overexpression experiments were performed by injecting 20 pg of *irx2a* cRNA into wild-type embryos. For *irx2a* and *irx2a*^*sa10776*^ rescue experiments, 20 pg of cRNA was co-injected with 133 µM *irx2a* MO. For *etv5a* rescue experiments, a combination of 220 pg *etv5a* cRNA with 133 µM *irx2a* MO was injected into wild-type embryos. For all rescue studies, replicate group sizes were a minimum of 30 embryos, and typically ranged between 40–60 embryos for each cohort.

### Quantification of phenotypes and statistical analysis

To measure domain length in the pronephros, embryos were mounted laterally in glycerol on a bridge slide. Either the polyline tool on the Nikon imaging software was used to trace the expression domain, or the segment line tool in FIJI. MCC cell number was assessed by viewing embryos dorsally at the highest magnification on a Nikon SMZ1000 stereomicroscope, and counting the individual *odf3b*^+^ cells in both nephrons^93^. Each experiments was completed in biological triplicate, with group sizes of at least n = 10 embryos, and confocal imaging was performed with group sizes of a least n = 3 representative embryos. ANOVA and student t-tests were used to assess statistical significance. Graphs were created using GraphPad Prism software.

### Chemical treatments

all-trans RA and DEAB (Sigma-Aldrich) were dissolved in 100% DMSO to make 1 M stock solutions, as previously described^16^. For all chemical treatments, wild-type embryos were incubated and protected from light in vehicle control, 1 × 10^−7^ M RA/DMSO or 1.6 × 10^−5^ M DEAB/DMSO made with E3 from 60% epiboly to 24 hpf. The chemical was then washed off and the embryos were fixed in 4% PFA. These chemical treatments were fully penetrant and produced consistent results over three replicates with n = 40–60 embryos per replicate analyzed for these studies.

## Supplementary information

Supplementary Information

## Data Availability

The data associated with this report are provided in the figures and supplemental figures.
