# Supplementary material for: Iroquois transcription factor irx2a is required for multiciliated and transporter cell fate decisions during zebrafish pronephros development
Source: Sci Rep. 2019 Apr 23;9:6454. doi: 10.1038/s41598-019-42943-y (PMC6478698; doi:10.1038/s41598-019-42943-y)

Iroquois transcription factor *irx2a* is required for multiciliated and transporter cell fate decisions during zebrafish pronephros development

Amanda N. Marra[1,2], Christina N. Cheng[1,2], Basma Adeeb[1], Amanda Addiego[1], Hannah Wesselman[1], Brooke E. Chambers[1], Joseph M. Chambers[1], and Rebecca A. Wingert[1,*]

[1]Department of Biological Sciences, Center for Stem Cells and Regenerative Medicine, Center for Zebrafish Research, University of Notre Dame, Notre Dame, IN 46556, USA

[2]Equal Contributions

[*]Corresponding Author

Correspondence: Rebecca A. Wingert, Ph.D., Department of Biological Sciences, University of Notre Dame, 100 Galvin Life Sciences, Notre Dame, IN 46556 USA; Email: rwingert@nd.edu; Phone: (574)-631-0907; Fax: (574)-631-7413

SUPPLEMENTAL FIGURE LEGENDS

Supplemental Figure 1: Validation of the *irx2a* splice blocking morpholino reagent. (A) The *IrxA* cluster is composed of *irx1a, irx2a,* and *irx4a* in zebrafish. The *irx2a* morpholino (MO) targets the splice acceptor site located at the 5' end of exon 2. F1 and R1 indicate the relative positions of the RT-PCR forward and reverse primers, respectively. Expected lengths of the wild-type and morphant RT-PCR bands are indicated in number of basepairs. (B) Aberrant splicing is confirmed in *irx2a* morphants generated by the *irx2a* splice MO. RT-PCR products are shown for the wild-type control and *irx2a* morphants. The green box indicates the wild-type bands, and the red asterisk indicates the morphant band. (C) Sequencing results of the wild-type and morphant bands present in *irx2a* morphants are displayed where the morphant band includes the intronic region between exon 1 and 2. (D) Predicted sequences of the wild-type and morphant Irx2a peptide. For the wild-type, alternating black and blue text color indicate sequence encoded by exon 1, 2, 3, and 4 in the open reading frame. By comparison, the morphant Irx2a peptide consists a normal peptide sequence based on exon 1 (black text underlined), followed by 16 aberrant amino acids (blue with yellow highlighting) followed by a premature stop. MO - morpholino , WT - wild-type, bp – basepairs.

Supplemental Figure 2: Nephron segmentation patterning is altered in *irx2a* morphants. (A) *irx2a* morphants are characterized by an expanded PST *(trpm7)*, a shifted DE *(slc12a1)*, and a reduced DL *(slc12a3)* by WISH. The PCT *(slc20a1a)* domain remains unchanged. The black bar illustrates the segment domain in relation to the somite number denoted above. Scale bar is 50 (μm). (B) Quantification of *slc20a1a* and (C) *trpm7* length. Each dot represents one nephron and data is presented +/-SEM. Significance was determined with an unpaired student's T-test. MO – morpholino.

Supplemental Figure 3: Standard control morpholino does not affect nephron segment or MCC development. (A) WISH was performed at the 28 somite stage for the PCT (*slc20a1a*), PST *(trpm7)*, DE *(slc12a1)*, DL *(slc12a3)* and MCC (*odf3b*) in uninjected controls and standard control morpholino injected wild-type zebrafish. Quantification of absolute segment length (B) and MCC number (C) revealed that there was no difference between these groups. Scale bar is 50 (μm). Each dot represents one nephron in a different embryo and data is presented +/-SEM. Significance was determined with an unpaired student's T-test. MO – morpholino.

Supplemental Figure 4: MCC loss in *irx2a* morphants continues at 36 hpf. (A) Dorsal view of the pronephros at 36 hpf where MCCs are labeled with *odf3b* in purple. Scale bar is 50 µm. (B) Quantification of *odf3b*+ cells at 36 hpf. Each dot represents one pronephros and data is presented +/-SEM. Statistical significance was determined via an unpaired student's T-test. MCC – multiciliated cell, hpf – hours post fertilization.

Supplemental Figure 1

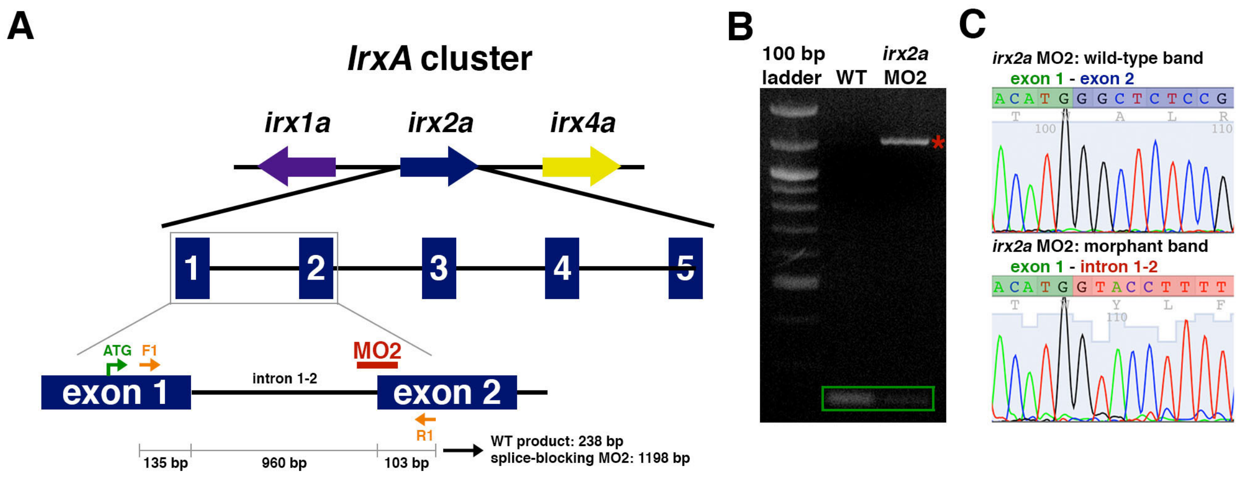

D

WT Irx2a predicted protein

MSYPQGYLYQPPGSLALYSCPLAAPRSEDLARSSSGSAFSPYPGSAAFTASANGFSSPLPYSTDPAT
GFPSYMGSPYDAHTTGMAGAISYHPYGSPGYPYQLNDPAYRKNATRDATATLKAWLQEHRKNPYPT
KGEKIMLAIITKMTLTQVSTWFANARRRLKKENKMTWAPRNKSEDEDEDDGDGERKDERTDKNMDN
SEASAEDEGISLHVDALTDHSCSVESDGEKVTCRTGDLVCDSGAEIQDKCEATDLGEERQRGASPKP
VTSSPLTGVEAPLLTHHHRENSTNKTCLDGQNQTVKPKLWSLAEIATSDPKQQNCPPGVGLLTSSAP
GASPAGAVYPATSILGRPLYYTSPFYSNYTNYGNFSPLQGQGILRYNSAAEGLLHKQTGEPLIKTNPN
QTEAHFRASNAESKKDPSEVFTVRSQSYLSSStop

MO2 Irx2a predicted protein

MSYPQGYLYQPPGSLALYSCPLAAPRSEDLARSSSGSAFSPYPGSAAFTASANGFSSPLPYSTDPAT
GFPSYMVPFQSRSLRIVLDRNAStop

Supplemental Figure 2

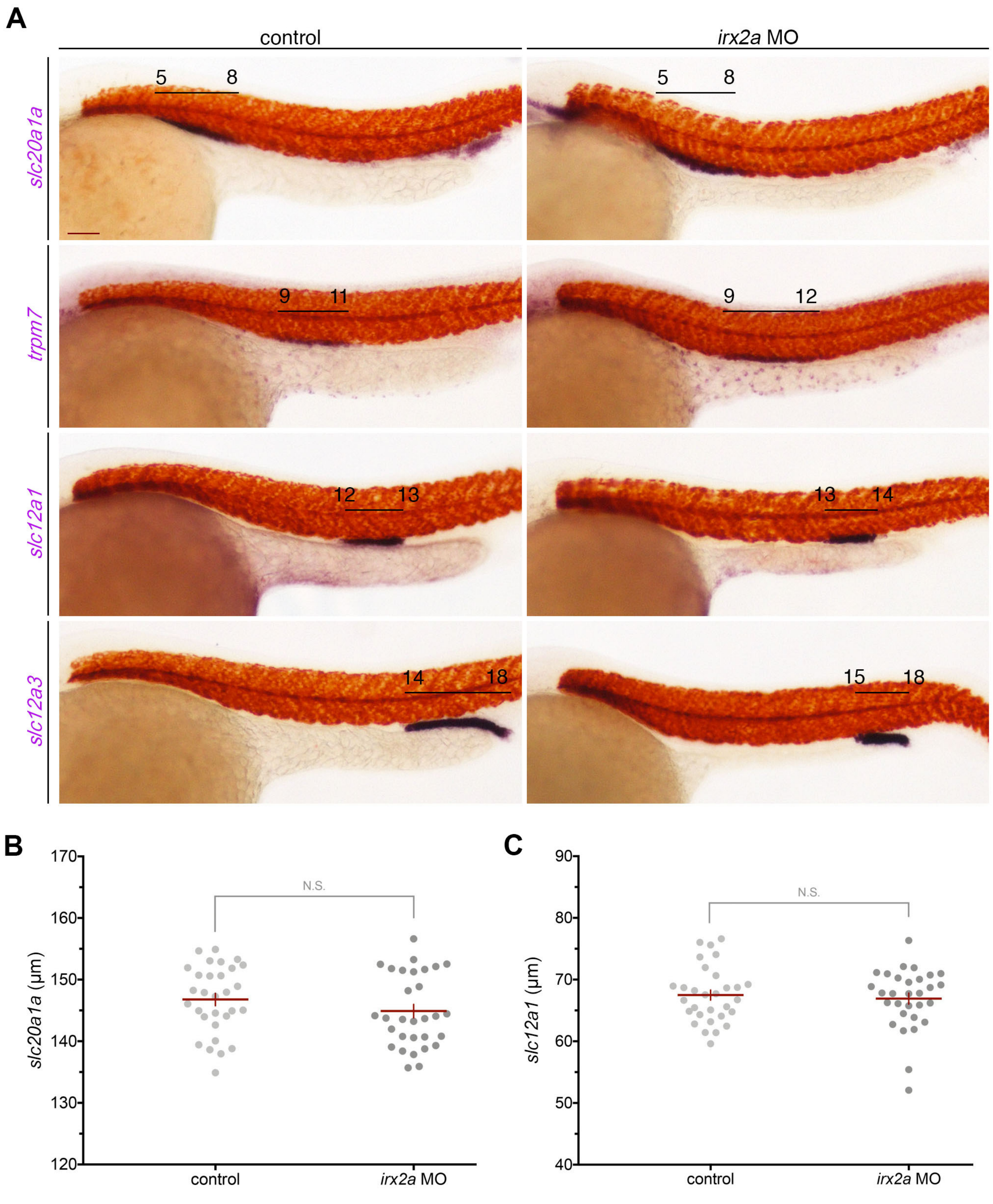

Supplemental Figure 3

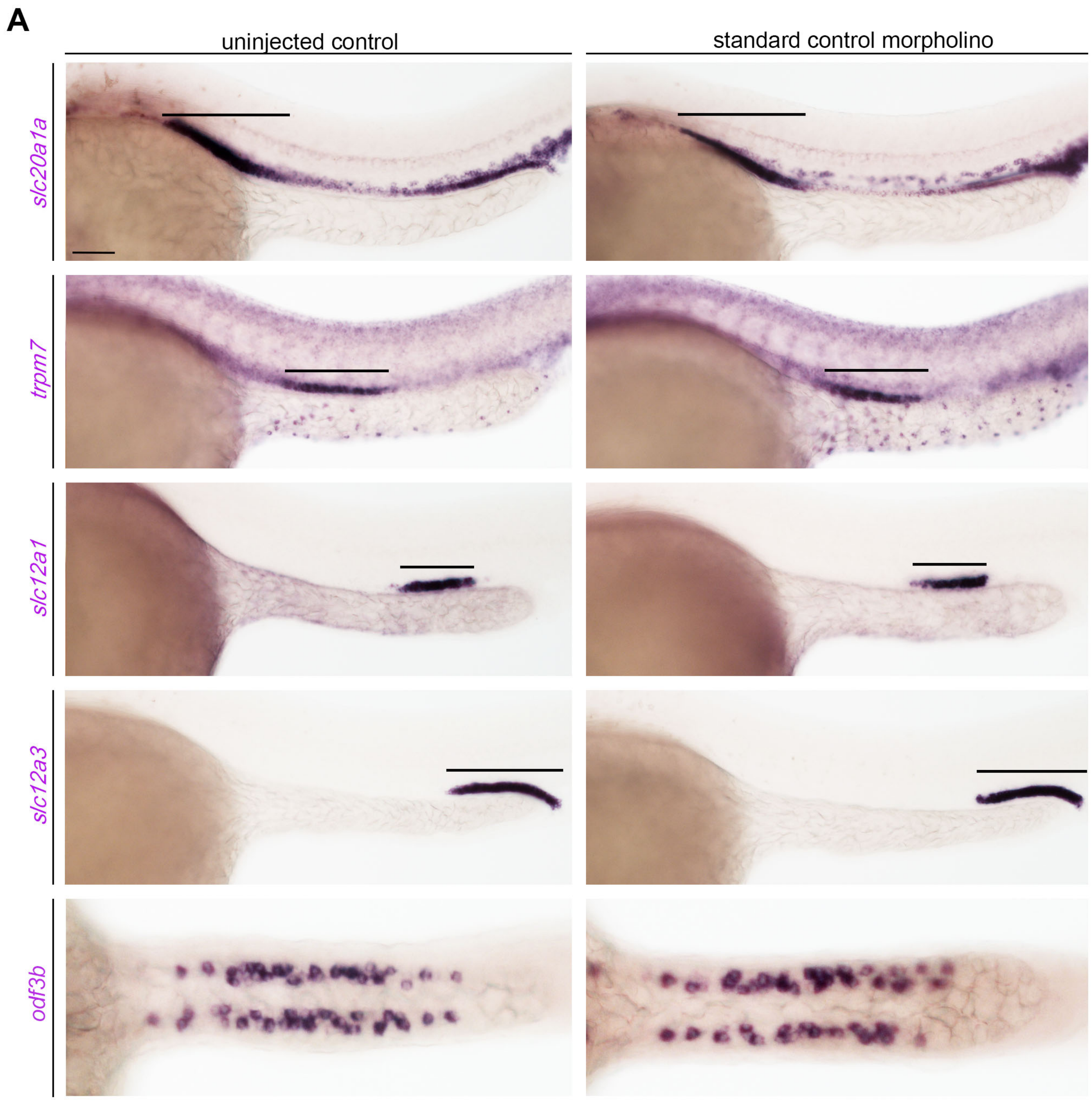

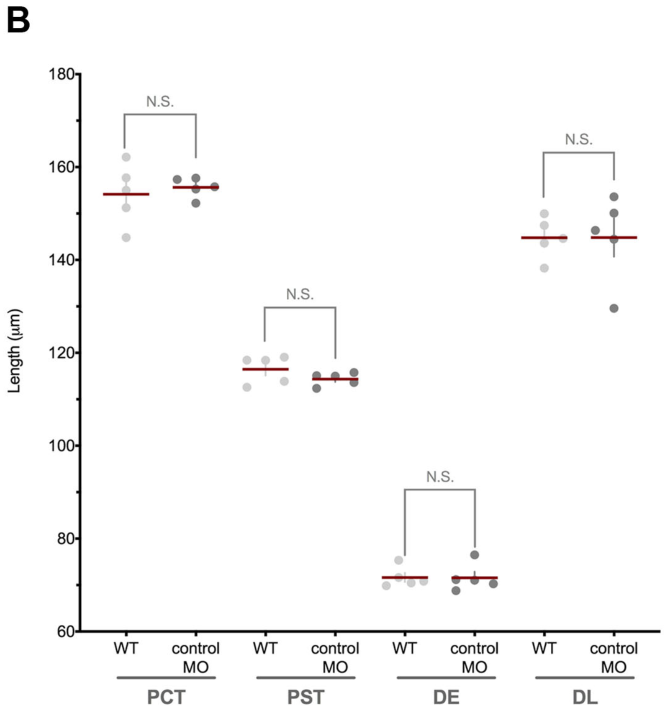

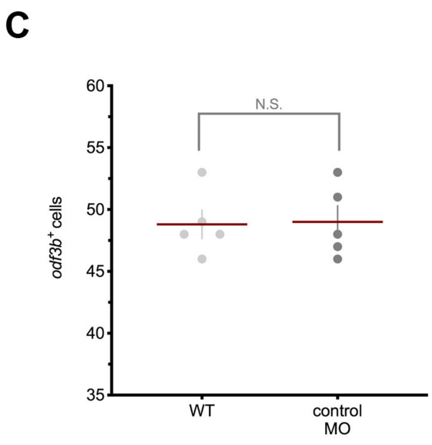

Supplemental Figure 4

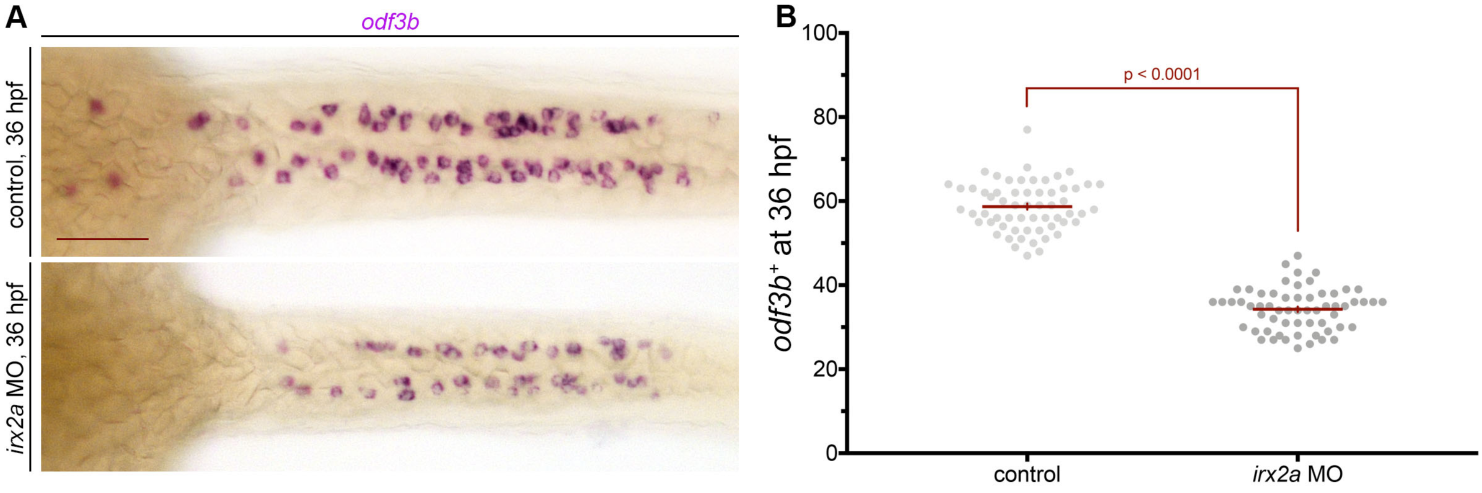

Supplement: Supplementary file 1 — Supplementary Information [file 41598_2019_42943_MOESM1_ESM.pdf]
